# Malaria in Pregnancy Interacts with and Alters the Angiogenic Profiles of the Placenta

**DOI:** 10.1371/journal.pntd.0003824

**Published:** 2015-06-19

**Authors:** Ricardo Ataíde, Oscar Murillo, Jamille G. Dombrowski, Rodrigo M. Souza, Flávia A. Lima, Giselle F. M. C. Lima, Angélica D. Hristov, Suiane C. N. Valle, Silvia M. Di Santi, Sabrina Epiphanio, Claudio R. F. Marinho

**Affiliations:** 1 Departamento de Parasitologia, Universidade de São Paulo (ICB/USP), São Paulo, São Paulo, Brazil; 2 Burnet Institute, Centre for Biomedical Research, Melbourne, Victoria, Australia; 3 Centro Multidisciplinar, Campus Floresta, Universidade Federal do Acre, Cruzeiro do Sul, Acre, Brazil; 4 Núcleo de Estudos em Malária, Superintendência de Controle de Endemias / Instituto de Medicina Tropical de São Paulo (IMT/USP), São Paulo, São Paulo, Brazil; 5 Departamento de Análises Clínicas e Toxicológicas, Universidade de São Paulo (FCF/USP), São Paulo, São Paulo, Brazil; Federal University of São Paulo

## Abstract

Malaria in pregnancy remains a substantial public health problem in malaria-endemic areas with detrimental outcomes for both the mother and the foetus. The placental changes that lead to some of these detrimental outcomes have been studied, but the mechanisms that lead to these changes are still not fully elucidated. There is some indication that imbalances in cytokine cascades, complement activation and angiogenic dysregulation might be involved in the placental changes observed. Nevertheless, the majority of studies on malaria in pregnancy (MiP) have come from areas where malaria transmission is high and usually restricted to *Plasmodium falciparum*, the most pathogenic of the malaria parasite species. We conducted a cross-sectional study in Cruzeiro do Sul, Acre state, Brazil, an area of low transmission and where both *P*. *vivax* and *P*. *falciparum* circulate. We collected peripheral and placental blood and placental biopsies, at delivery from 137 primigravid women and measured levels of the angiogenic factors angiopoietin (Ang)-1, Ang-2, their receptor Tie-2, and several cytokines and chemokines. We measured 4 placental parameters (placental weight, syncytial knots, placental barrier thickness and mononuclear cells) and associated these with the levels of angiogenic factors and cytokines. In this study, MiP was not associated with severe outcomes. An increased ratio of peripheral Tie-2:Ang-1 was associated with the occurrence of MiP. Both Ang-1 and Ang-2 had similar magnitudes but inverse associations with placental barrier thickness. Malaria in pregnancy is an effect modifier of the association between Ang-1 and placental barrier thickness.

## Author Summary

*Plasmodium* infections during pregnancy represent a substantial health burden for mothers and their neonates leading to underweight babies, preterm deliveries, abortions, stillbirths, and even maternal mortality. The placenta, in its role as a bridge between mother and foetus, will either suffer or mediate the injuries which eventually lead to these adverse outcomes. *Plasmodium* infections during pregnancy can affect the metabolism of the placenta, its functions and even its architecture. These changes are well described in Sub-Saharan Africa, where *P*. *falciparum* infections predominate, but little is known in areas where *P*. *vivax* is predominant. The state of Acre in Brazil is one such area. We have recently shown that malaria in pregnancy is associated with placental pathology in this region. In the present study we sought to find out the association between the placental and peripheral levels of angiogenic factors, which are known and important players in the formation and function of the placenta, and the occurrence of placental pathology in *Plasmodium*-infected women. We found that, lower levels of Angiopoietin-1 in women who experienced malaria during pregnancy were associated with specific modifications occurring in the placenta. This provides a possible pathway through which these pathological changes occur.

## Introduction

In countries endemic for *Plasmodium* spp., malaria in pregnancy (MiP) remains an important health burden that can result in maternal anaemia, small-for-gestational-age babies, low birth weight, and even miscarriages and stillbirths [1–3]. Most of the studies looking at possible mechanisms for these outcomes have been conducted in areas where *P*. *falciparum* is either the only parasite species present or the main species in circulation.

Some of the associations found with these outcomes, which are not necessarily mutually exclusive, include the sequestration of parasites in the placenta, the presence of monocytes and inflammatory cytokines in the placenta, activation of the complement cascade, dysregulation of hormonal and glucose pathways, compromised amino acid uptake by the placenta and impaired angiogenesis [4–11]. Some of the inflammatory cytokines associated with MiP include (tumour necrosis factor (TNF)-α, interferon (INF)-γ), interleukin (IL)-10, IL-8, IL-6 and macrophage inflammatory protein (MIP)-1α [12–17]. The angiogenic factors angiopoietin (Ang)-1, Ang-2 and Tie-2 (which serves as a receptor for both Ang-1 and Ang-2) are involved not only in vasculogenesis and angiogenesis but also play distinct roles in mediating inflammation in infectious diseases [8,18–21].

Despite the fact that *P*. *vivax* infections can also result in severe outcomes for both mother and child, there are relatively few studies conducted in areas where *P*. *vivax* is the most prevalent *Plasmodium* species [22–24]. The Amazon region, in Brazil, is one such area. We are beginning to understand some of the basic pathological processes associated with *P*. *vivax* during pregnancy and have recently shown that the placentas of women exposed to *P*. *vivax* during pregnancy have increased barrier thickness, syncytial knots and monocyte numbers compared to uninfected placentas, even in the absence of evidence for parasite sequestration (a hallmark for *P*. *falciparum-*associated pathology) [25]. What contributes to these histopathological observations is not known. Other studies have observed modifications in the vasculature within the placental villi suggesting that this parasite impacts angiogenic processes [26].

In this study we aimed to measure plasma levels of angiogenic factors and cytokines in women exposed to *Plasmodium* spp. during pregnancy in an area where *P*. *vivax* is predominant and to associate those levels with pathological features of the placenta.

## Materials and Methods

### Study design, region and population

The region where our study was conducted has been described elsewhere. This region presents a higher prevalence of *P*. *vivax* infections than *P*. *falciparum* infections [27]. A cross-sectional study of primigravidae at delivery was conducted in the maternity unit of the Hospital da Mulher e da Criança do Juruá in Cruzeiro do Sul, Acre, Brazil from December 2012 to August 2013. A study team member approached every eligible woman who arrived at the maternity and questionnaires were applied to every woman who agreed to participate in the study to obtain epidemiological data. Women with a history of smoking during pregnancy, drug use and who presented with syphilis, HIV or hepatitis were excluded from the study. Due to the extremely high percentage of C-sections performed in Brazilian maternity units, women who underwent a C-section were not excluded from the study; however this was controlled for in the analysis.

### Collection and processing of samples

Peripheral blood and placental blood (from the maternal side of the placenta) were collected in heparin tubes. Thin and thick smears were made and stained with Giemsa, and two drops of blood were spotted on filter paper for assessment of malaria status by Nested-PCR. Biopsies of placental tissue were collected, fixed in 10% neutral buffered formalin at 4°C and then kept in ethanol 70% until they could be sent to São Paulo University for processing. Paraffin-embedded sections of placental tissue were stained with Haematoxilin-Eosin (H&E) or Masson’s Trichrome Stain (MTS) for histological examination. A Zeiss Axio Imager M2 light microscope equipped with a Zeiss Axio Cam HRc camera was used to capture images of the placentas. Some of the parameters were evaluated and analysed using Image J (Image J 1.46c, Wayne Rasband, National Institutes of Health, USA, http://imagej.nih.gov/ij). Additionally, medical data was collected concerning blood-pressure, haemoglobin and haematocrit levels and axillary temperature.

### Evaluation of malaria infection

Malaria during pregnancy was diagnosed by microscopy by the endemic surveillance team of Cruzeiro do Sul, Acre, Brazil. Malaria at delivery was diagnosed by evaluation of a peripheral/placental blood smear and/or by molecular methods (nested-PCR). For molecular detection, DNA was obtained from dried-blood spots (placental and peripheral) on filter paper through the use of a commercially available extraction kit (QIAmp DNA Micro, Qiagen), following the manufacturer’s instructions. The nested-PCR reaction was conducted as previously published by dos Santos et al. [28].

### Evaluation of histopathology

Histopathological parameters were analysed using the Tissue Microarray technique, conducted at the AC Camargo Hospital, with the exception of the evaluation of immune cells, which was performed on slides containing the full length of the tissue. Two individuals performed all measurements. Cases that proved to be contradictory between observers were re-evaluated until consensus was reached. All histological evaluation methods were optimised by our group and are described elsewhere [25].

### Measurement of angiogenic factors by ELISA

The angiogenic factors angiopoietin (Ang)-1 (1:20 dilution) and Ang-2 (1:10 dilution) and their associated soluble receptor Tie-2 (1:20 dilution), were measured using the commercially available DuoSet ELISA development kits from R&D systems, according to the manufacturer’s instructions.

### Measurement of cytokines by bead array

Levels of cytokines in both placental and peripheral plasma were measured with the commercially available Millipore kit HCYTOMAG-60K-07 (IL-1β, IL-10, IL-6, IL-8, MIP-1α, TNF-α), using Luminex technology and following the manufacturer’s instructions. All plasma samples were processed and kept at -80°C in Cruzeiro do Sul until they were sent to University of São Paulo. All available samples of placental blood were evaluated while for peripheral blood a random subset of uninfected women (n = 12) and infected women (n = 28) were chosen.

### Definitions

Malaria in pregnancy was defined as evidence of *Plasmodium* infection during pregnancy or at term by microscopy. Current infection was defined as a *Plasmodium* infection detected at term by microscopy and/or histology and/or PCR. Anaemia was defined as a haemoglobin level lower than 11 g/dL. Low birth weight was defined as an infant weight of less than 2,500 g.

### Statistical analysis

Data were analysed using Stata 12 software (StataCorp, College Station, TX, USA) and GraphPad Prism (GraphPad Prism version 5 for Mac OX, GraphPad Software, San Diego CA, USA, www.graphpad.com). Variables with normal distributions were analysed using the means and standard deviation, and the variables that were non-normally distributed were analysed using the medians and interquartile range. Differences in the mean values between groups were evaluated using Student’s t-tests or Mann-Whitney U-tests accordingly. Categorical data and proportions were analysed using chi-square tests. All placental parameters evaluated were ln-transformed before statistical analysis was performed. For determining the effects of one or more infections on the histopathological parameters measured and MiP infection status on angiogenicfactors/cytokines one-way ANOVA tests with Bonferroni correction were used. Multivariate linear regressions with placenta parameters as outcome variables were used to look for associations with levels of angiogenic factors and cytokines. Gestational age and C-section were found to be confounders and were included in the models. To uncover the role of *Plasmodium* spp. infection during pregnancy as an effect modifier of the association between angiogenic factors and cytokines with placental parameters, we introduced an interaction term in the multilinear regression model.

### Ethics considerations

Ethical clearance was provided by the committees for research of the University of São Paulo and the Federal University of Acre (Plataforma Brasil, CAAE: 05736812.0.0000.5467 and 05736812.0.3001.5010, respectively). All the study participants gave written informed consent or had their legal guardians do so, if they were minors.

## Results

### Study samples and population

During the eight months of recruiting, 147 women were enrolled into the study. Of those, 10 women were excluded from the analysis due to use of cigarettes and/or illicit drugs (7 women) during pregnancy or presence of infections (syphilis, hepatitis B, hepatitis C or HIV). The present study comprised 137 women (Table 1).

**Table 1:** Characteristics of study subjects by infection status.

		**Uninfected** ^9^		**Malaria in Pregnancy** ^9^		***p*-value** ^10^
			n		n	
****Age (years), mean (SD)****		19.7 (4.2)	92	18.5 (2.7)	45	0.064
****Rural residence**** ^1^ ****(%)****		45.0	41	56.0	26	0.205
****Welfare (%)****		35.2	32	43.5	20	0.344
****ANC visits**** ^2^		46.0	40	16.0	39	0.367
****Hematocrit (%), mean (SD)****		35.8 (4.4)	75	35.1 (3.8)	39	0.338
****Haemoglobin (g/dL), mean (SD)****		11.8 (1.4)	77	11.6 (1.3)	39	0.521
****Anaemia**** ^3^ ****(%)****		26.0	20	28.2	11	0.798
****Axillary temperature (°C), mean (SD)****		36.2 (0.4)	86	36.7 (1.2)	42	**<0.001**
****Blood pressure (mmHg), mean (SD)****	Systolic	119.3 (10.5)	86	115.5 (12.5)	42	0.072
	Diastolic	77.5 (7.6)	86	75.0 (10.7)	42	0.112
****Gestational age (weeks), mean (SD)****		39.0 (2)	91	39.1 (1.8)	43	0.947
****Premature**** ^4^ ****(%)****		6.6	6	7.0	3	0.934
****C-section****		52.1	38	40.5	17	0.231
****Birth weight (g), mean (SD)****		3224.0 (513.9)	80	3102.1 (453.5)	43	0.194
****Low birthweight**** ^5^ ****(%)****		7.5	6	7.0	3	0.915
****Malaria before gestation**** ^6^ ****(%)****		60.9	56	88.9	40	**0.001**
*****Plasmodium*****spp. during pregnancy**** ^7^:						
*****Plasmodium vivax (%)*****		NA	NA	13.9	19	---
*****Plasmodium vivax,*****delivery**** ^8^ *****(%)*****		NA	NA	21.1	4	---
*****Plasmodium falciparum*****(%)****		NA	NA	10.2	14	---
*****Plasmodium falciparum,*****delivery**** ^8^ ****(%)****		NA	NA	35.7	5	——

^1^As reported by the patient.

^2^Antenatal care visits. Women are encouraged to attend 7 or more ANC visits by the government. If they do so, they receive a pre-natal bundle.

^3^Defined as <11 g/dL of hemoglobin.

^4^Defined as a gestational age <37 weeks.

^5^Defined as a birthweight <2500 g.

^6^As reported by the patient.

^7^Numbers represent women with single species infections. Nine women in total (included in the malaria in pregnancy group) had either sequential or simultaneous infections with both species. Three women had infections that were not identified.

^8^Species identified using microscopy and/or nested-PCR done on either peripheral or placental blood at delivery.

^9^Defined by the microscopic examination of slides during gestation and/or the presence of *Plasmodium* spp. or *Plasmodium* spp. products identified by histology or PCR after delivery.

^10^Differences in the mean values between groups were evaluated using Student’s t-tests. Categorical data and proportions were analysed using Chi-square test.

A total of 92 out of the 137 women were defined as uninfecteds after microscopic, histologic or molecular examination revealed no evidence of *Plasmodium* spp. infection either during pregnancy or at delivery. The women without evidence of *Plasmodium* infection tended to be older than the women who presented an infection during pregnancy [mean (standard deviation (SD)]: 19.7 (4.2) vs 18.5 (2.7), *p* = 0.064, Table 1. Clinically, both groups of women seemed to differ only in the axillary temperature (uninfected vs infected, mean (SD): 36.2 (0.4) vs 36.7 (1.2), *p*<0.001, Table 1). Interestingly, both groups of women had similar levels of haemoglobin at delivery. The babies from both groups were also similar in terms of gestational age at delivery and birth weight (Table 1). A history of malaria prior to pregnancy was more often reported by those in the infected group vs women in the uninfected group (88.9% vs 60.9%, *p* = 0.001, Table 1).

**Fig 1:**
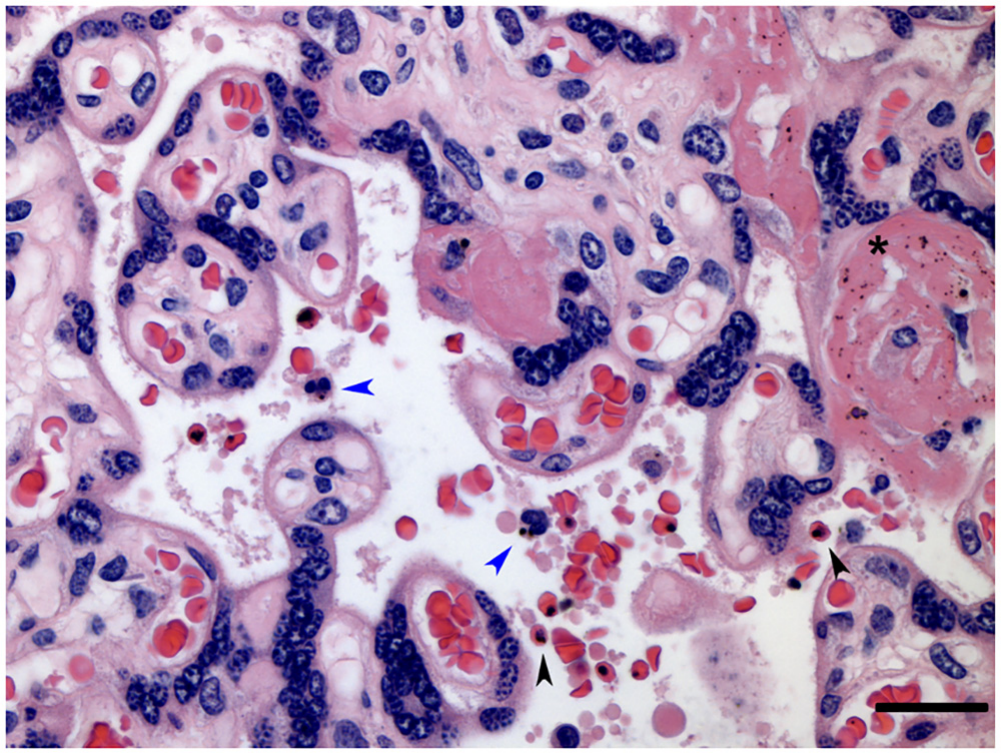
Histological evidence of *P*. *falciparum* in the placenta of nested PCR-positive women at delivery. Representative image of a placenta with positive nested-PCR for *Plasmodium falciparum* at delivery. Bar represents 50 μm. Gray Black arrowheads indicate presence of infected erythrocytes. Blue arrowheads indicate leucocytes with parasite pigment. Asterisk indicates parasite pigment deposited in fibrinoid tissue. 400x magnification.

A total of 19 women were identified as having had *P*. *vivax*-only infections during pregnancy. Twenty-one percent of those women presented with infections at delivery (Table 1), with no detectable parasites in the placenta. *P*. *falciparum* infections accounted for a third (n = 14) of the single species infections detected during pregnancy, with 5 women showing infections at delivery (Table 1). Of these 5 women, two showed abundant evidence of parasite and parasite pigment in the placenta (Fig 1).

### Placental parameters and histopathology

The placentas of the women who experienced a *Plasmodium* infection during pregnancy did not differ in weight from those who remained uninfected (Table 2). Similarly, there was no difference between both groups of women regarding parameters of placental histopathology (syncytial knots, thickness of the placental barrier and presence of monocytes) (Table 2). However, when the women who experienced more than one infection during their pregnancy were compared to uninfected women, there was a small but significant increase in both the thickness of the placental barrier (median [IQR]) 4.52 [4.25, 5.12] vs 4.17 [3.56, 6.61], *p* = 0.023 and the percentage of intervillous monocytes 3.13 [2.19, 4.22] vs 2.02 [1.20, 3.12], *p* = 0.025 (Table 2). Despite the small numbers, it is possible to realise that the increase in the thickness of the placental barrier observed is at least partially driven by *P*. *vivax*, while the increase in the percentage of intervillous monocytes seems independent of it (S1 Fig).

**Table 2:** Placental and histological parameters according to malaria status and number of infections^1^.

	**Uninfected**	**MiP (total)** ^**2**^	***p*-value**	**More than 1 infection** ^**3**^	***p*-value**
Placental weight (g)	557.1 [488.80, 620.00]	525.9 [492.95, 597.25]	0.374	521.6 [488.1, 579.0]	0.312
Syncytial knots (%)	10.0 [8.0, 14.25]	11 [6.5, 14.5]	0.799	10.5 [7.5, 12.5]	0.789
Barrier thickness (μm)	4.17 [3.56, 6.61]	4.11 [3.61, 4.58]	0.936	4.52 [4.25, 5.12]	**0.023**
Monocytes (%)	2.02 [1.20, 3.12]	2.22 [1.14, 3.14]	0.793	3.13 [2.19, 4.22]	**0.025**

^1^Data were not normally distributed, and the medians with interquartile ranges are presented. Differences between uninfected and MiP (total) groups were evaluated using Mann-Whitney rank sum tests. Differences between uninfected, one infection and more than 1 infection groups were evaluated by one-way ANOVA with Bonferroni correction. All *p*-values refer to differences between that group and the uninfected group (n = 84).

^2^Includes all women diagnosed microscopically and/or molecularly with a *Plasmodium* spp. infection during pregnancy (n = 44).

^3^Women with more than one *Plasmodium* spp. infection diagnosed during pregnancy (microscopically and/or molecularly) (n = 16).

### Placental and peripheral levels of angiogenic factors and cytokines

**Fig 2:**
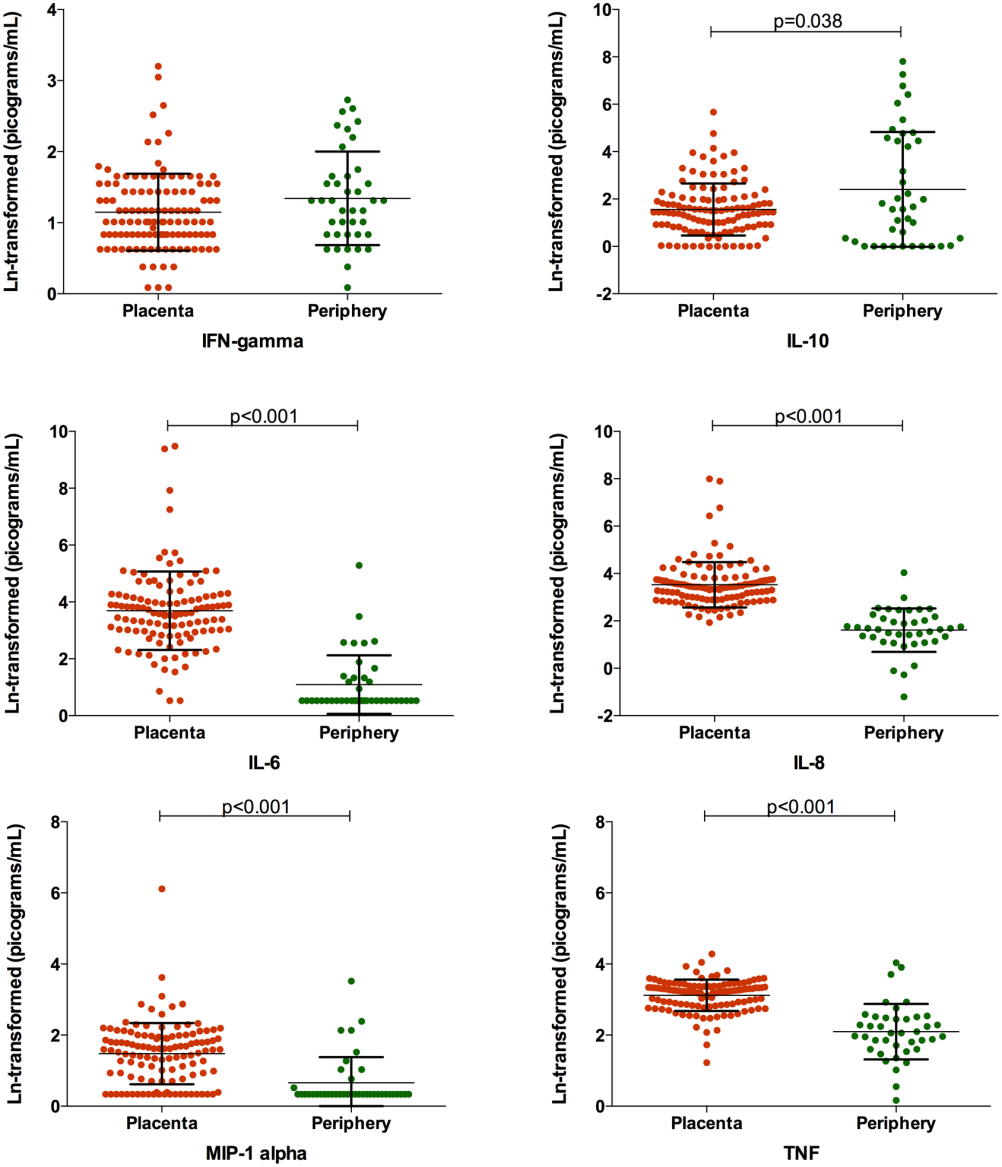
Placental and peripheral blood cytokine levels. Placental (orange, n = 118) and peripheral (green, n = 40) levels of cytokines measured in this study. All levels are ln-transformed in picograms/mL. Bars represent the mean and standard deviation. Student’s t-tests with Welch correction were performed to evaluate differences between the two compartments.

**Fig 3:**
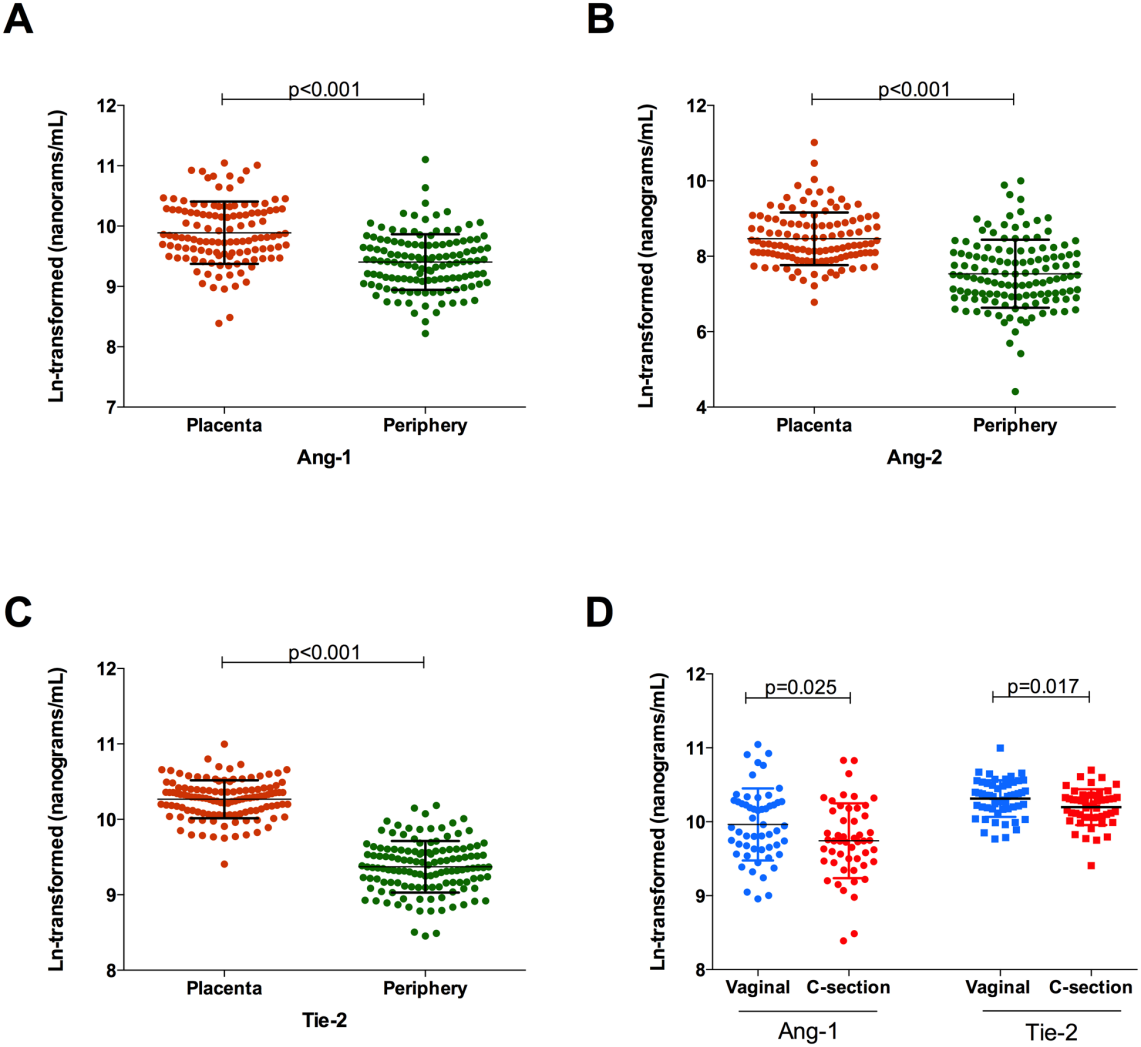
Placental and peripheral blood levels of angiogenic factors and effect of delivery method. (A-C) Placental (orange, n = 121) and peripheral (green, n = 130) levels of angiogenic factors measured in this study. (D) Placental blood levels of Ang-1 and Tie-2 in women who had vaginal deliveries (blue, n = 54) or who underwent C-sections (red, n = 51). All levels are ln-transformed in nanograms/mL. Bars represent the mean and standard deviation. Student’s t-tests with Welch correction were performed to evaluate differences between the two compartments.

Levels of cytokines and chemokines (with the exception of IFN-γ and IL-10) were found to be higher in the placenta than in the periphery, although this result should be taken with caution due to the small number of samples evaluated (Fig 2). Similarly, the levels of Ang-1, Ang-2 and Tie-2 were increased in the placenta compared to the periphery (Fig 3A, 3B and 3C). Contrary to what was observed for cytokine levels, the levels of Ang-1 and Tie-2 were significantly reduced (*p* = 0.025 and *p* = 0.017 respectively) in those women who underwent a caesarean (Fig 3D), possibly reflecting the nature of the hormonal changes that occur during the normal course of a vaginal delivery.

Placental and peripheral levels of both cytokines and chemokines did not generally vary between women with *Plasmodium* spp. infection during pregnancy and those without (Table 3). Similarly, placental levels of angiogenic factors were also identical between the women with *Plasmodium* spp. infection during pregnancy and those without (Table 3). However, the women with evidence of a current malaria infection had increased levels of cytokines in the periphery and of IL-10 in the placenta, which was also seen in vivax-only infected women (Table 3 and S2 and S3 Figs). Additionally, we observed a decrease of peripheral Ang-1 in women with a current infection and a significantly higher ratio of Tie-2:Ang-1 in both the total women who had MiP or only the women who had a current infection, relative to the uninfected women (Table 3). Though the numbers are small, if we segregate current malaria in pregnancy by *P*. *vivax* and *P*. *falciparum* the data shows that both the decrease of Ang-1 and the increase in the ratio of Tie-2:Ang-1 are more closely associated with *P*. *falciparum* than with *P*. *vivax* (Ang-1 (median [min, max]): Uninfected (n = 88): 12.67 [3.71, 32.16], vivax (n = 4): 9.19 [9.14, 9.85], *p* = 0.975, falciparum (n = 5): 8.89 [8.56, 9.51], *p* = 0.098; ratioTie2:Ang-1: Uninfected (n = 88): 0.89 [0.20, 2.56], vivax (n = 4): 0.76 [0.50, 1.51], *p* = 0.766, falciparum (n = 5): 1.42 [0.86, 2.65], *p* = 0.096).

**Table 3:** Levels of cytokines, chemokines and angiogenic factors by compartment and malaria in pregnancy status^1^.

		**Uninfected**	**MiP (total)** ^**2**^		**MiP (current infection)** ^**3**^	
**Periphery**	**Angiogenic factors (ng/mL)**	**Median [IQR]**	**Median [IQR]**	***p*-value**	**Median [IQR]**	***p*-value**
	Angiopoietin-1	12.67 [9.21, 17.52]	11.29 [7.62, 13.72]	0.284	9.33 [6.23, 12.89]	0.058
	Angiopoietin-2	1.75 [1.09, 3.29]	1.89 [0.92, 3.20]	0.589	2.21 [0.86, 3.20]	0.886
	Tie-2	11.61 [10.63, 14.94]	11.63 [10.63, 14.94]	0.322	13.7 [9.46, 15.31]	0.617
	Ratio Tie-2/Angiopoietin-1	0.89 [0.67, 1.20]	1.18 [0.82, 1.53]	**0.043**	1.14 [0.86, 1.64]	**0.013**
	**Cytokines (pg/mL)**	**Median [IQR]**	**Median [IQR]**	***p*-value**	**Median [IQR]**	***p*-value**
	IFN-γ	3.22 [2.30, 4.72]	3.72 [2.87, 4.71]	0.117	4.47 [2.99, 11.85]	**0.013**
	IL-10	1.62 [1.01, 6.02]	8.41 [1.73, 131]	**0.046**	175 [53.11, 740]	**<0.001**
	IL-6	1.7 [1.70, 2.14]	1.7 [1.7, 4.65]	0.392	2.74 [1.70, 12.98]	0.122
	IL-8	4.8 [3.96, 7.84]	5.49 [3.09, 10.63]	0.742	8.34 [5.58, 12.26]	**0.042**
	MIP-1α	1.4 [1.40, 1.40]	1.4 [1.40, 2.48]	0.127	2.11 [1.40, 8.47]	**0.018**
	TNF-α	6.39 [3.72, 8.68]	9.39 [6.24, 12.94]	0.090	14.11 [9.85, 29.70]	**0.011**
**Placenta**	**Angiogenic factors (ng/mL)**	**Median [IQR]**	**Median [IQR]**	***p*-value**	**Median [IQR]**	***p*-value**
	Angiopoietin-1	18.56 [14.22, 29.27]	18.03 [12.35, 27.41]	0.139	17.31 [10.31, 29.43]	0.269
	Angiopoietin-2	4.19 [3.19, 7.25]	3.97 [2.69, 7.49]	0.358	3.38 [3.08, 7.02]	0.566
	Tie-2	29.27 [24.67, 33.41]	29.53 [23.99, 32.41]	0.308	28.28 [23.73, 36.59]	0.440
	Ratio Tie-2/Angiopoietin-1	1.36 [0.99, 2.14]	1.37 [1.01, 2.04]	0.368	1.12 [0.94, 2.93]	0.639
	**Cytokines (pg/mL)**	**Median [IQR]**	**Median [IQR]**	***p*-value**	**Median [IQR]**	***p*-value**
	IFN-γ	2.75 [2.30, 4.20]	2.99 [2.30, 4.97]	0.287	3.22 [2.3, 6.28]	0.083
	IL-10	4.25 [2.05, 7.82]	4.25 [2.40, 6.70]	0.657	5.05 [3.48, 23.93]	**0.011**
	IL-6	44.29 [21.03, 66.39]	26.78 [17.36, 72.30]	0.462	36.51 [18.04, 142.00]	0.954
	IL-8	31.08 [19.05, 41.85]	28.41 [17.81, 47.66]	0.706	29.23 [17.92, 48.64]	0.749
	MIP-1α	4.87 [2.15, 7.31]	4.9 [2.41, 6.74]	0.944	3.96 [2.55, 6.66]	0.669
	TNF-α	25.12 [16.86, 28.63]	26.25 [18.49, 30.26]	0.449	25.19 [17.67, 28.98]	0.962

^1^Data were not normally distributed, and the medians with interquartile ranges are presented. Differences between uninfected and MiP (total) groups were evaluated on ln-transformed data using Student’s t-tests with Welch correction. Differences between uninfected, MiP (past infection) and MiP (current infection) groups were evaluated by one-way ANOVA with Bonferroni correction. All *p*-values refer to differences to the uninfected group (n = 12).

^2^Includes all women diagnosed microscopically and/or molecularly with a *Plasmodium* ssp. infection during pregnancy (n = 40).

^3^Women with confirmed *Plasmodium* ssp. infection at delivery (microscopically and/or molecularly) (n = 12).

### Association between angiogenic factors and cytokines with placental histological parameters

**Fig 4:**
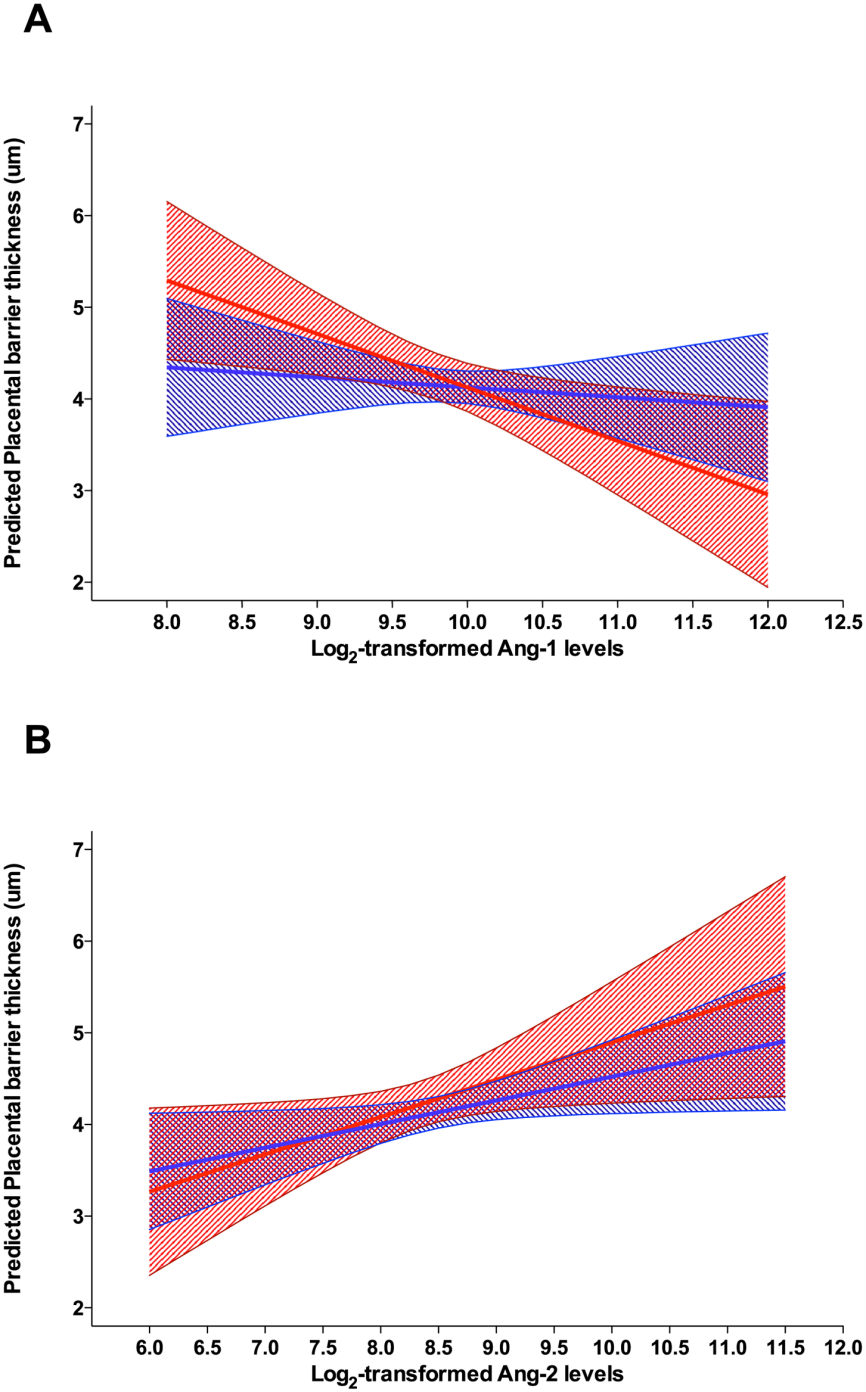
Evaluation of the role of malaria during pregnancy as an effect modifier of the association between placental barrier thickness and placental angiopoietin-1 levels. A linear model of the association between the levels of both Ang-1 (A) and Ang-2 (B) with thickness of the placental barrier was evaluated with the inclusion of an interaction term between the occurrence of malaria during pregnancy and levels of angiogenic factors. The mean predicted values and 95% confidence intervals for placental barrier thickness (y-axis) are plotted for uninfected women (blue) and women with malaria during pregnancy (red line). There is graphical evidence of a modification effect of malaria during pregnancy on the association of Ang-1 and thickness of the placental barrier (p for interaction = 0.109), but this graphical evidence is not clearly perceptible for Ang-2 (p for interaction = 0.507).

Associations between levels of angiogenic factors and cytokines with placental parameters were assessed for factors and cytokines measured in the placenta. Multilinear regression models, controlling for possible confounders (caesarean and gestational age) (S1 Table) revealed similar strength but opposite associations between placental barrier thickness and increased levels of Ang-2 (coeficient [95% CI], *p*-value) 0.30 [0.10, 0.51], *p* = 0.004 and Ang-1–0.30 [-0.60, -0.01], *p* = 0.045. Increased Ang-1 was also associated with a decrease in placental weight -36.34 [-69.76, -2.91], *p* = 0.033. Rather then segregating the women into MiP positive or negative, thus reducing the power of our analysis, we investigated whether these associations were modified by the presence or absence of malaria during pregnancy by adding *Plasmodium* spp. infection as an interaction term in the model. Interestingly, the association between Ang-2 and placental barrier thickness was not modified by the presence of *Plasmodium* infection; however, there was some evidence that malaria during pregnancy altered the effect of Ang-1 on placental barrier thickness (Fig 4).

## Discussion

In this study, we were able to measure and compare cytokine and angiogenic factors levels at delivery between women who had malaria during pregnancy and those who did not. Additionally, we associated the levels of these molecules with the occurrence of histopathological changes in the placenta.

Malaria during pregnancy in this region of Brazil has previously been associated with detrimental outcomes for both mother and child [25,29–31]. The placenta, in its role as a bridge between mother and foetus, will either suffer or mediate some of the injuries to both the mother and the foetus, which eventually lead to these adverse outcomes [32,33]. In Africa, where virtually all malaria infections are caused by *P*. *falciparum*, the placental events and mechanisms that contribute to adverse obstetric outcomes have been extensively studied and include adhesion of the parasite to the placenta [4], accumulation of inflammatory cells with production of cytokines [5], histological modifications to the villi [6], complement activation [7–9], disruption of nutrient transport and disturbance of the angiogenesis process [10,11]. In areas where *P*. *vivax* is also present, both *P*. *falciparum* and *P*. *vivax* infections are able to significantly impact placental development and foetal outcomes [34–36], but the mechanisms involved have been insufficiently studied [26]. Because both our recently published results and those of others show that *P*. *vivax* and *P*. *falciparum* seem to have a similar magnitude of effects on the placenta in areas of low malaria transmission and where treatment is readily available [25,26], we grouped all *Plasmodium* spp. infections together for evaluation.

In this study, the *Plasmodium-*infected and uninfected women had very similar epidemiological characteristics. Additionally, apart from an increase in the axillary temperature in those who were infected, no other clinical features were significantly different between the groups of women, including haemoglobin levels, prevalence of anaemia, newborn birth weight and proportions of prematurity and low birth weight. These results may be a positive reflection of the active and prompt diagnosis and treatment policies in place in Acre state, where our study site is located and where women are often diagnosed and treated under supervision within 48 h [37].

Placental parameters between infected and uninfected women differed when the number of infections during pregnancy was taken into account. Consistent with our previous report [25], the women who experienced more than one infection during pregnancy had significantly increased placental barrier thickness and mononuclear cells in the intervillous space, compared to uninfected women. This finding highlights the accumulation of insults to the placenta when multiple infections occur.

The observation that placental levels of both cytokines and angiogenic factors were significantly higher in the placenta than in the periphery, independently of malaria infection status, may reflect the different time-points of collection of samples (peripheral blood collected before delivery; placental blood collected after delivery) but is also a clear indication of the distinct nature of both these compartments. When studying placental parameters, one should focus on the placental milieu and not rely on that of the periphery.

Similar to research findings described in other populations [11,38], we observed a decrease in peripheral Ang-1 levels in the pregnant women with an active infection at delivery as well as an increase in the ratio between Tie-2:Ang-1 in the women who had malaria during pregnancy. Interestingly, this seemed to be associated mainly to *P*. *falciparum* infections. Neither the level of placental Ang-1 nor the placental ratio Tie-2:Ang1 were different between women with and without infection. This may be a reflection of the low grade placental infections or, most likely, it may reflect different regulatory mechanisms in the peripheral and the placental compartments regarding the levels of these factors. Peripheral levels of all cytokines and chemokines (with the exception of IL-6) were increased in the women who had malaria at delivery, and this appears to be true for those women with *P*. *vivax* infection only, but the low numbers from whom these levels were available do not allow for a more substantial analysis. In the placenta, levels of IL-10 were significantly increased in the women who had malaria at delivery compared to the uninfected women. Once more, low numbers dictated the analysis but women with *P*. *vivax* only also appeared to have higher levels of placental IL-10. This finding substantiates the role of IL-10 not only as a marker of MiP but of malaria infection in general [39].

Increased levels of Ang-1 were associated with decreases in placental weight and with a decrease in placental barrier thickness in the entire cohort of patients. An analysis of the effect of malaria during pregnancy on this association revealed that the burden of the interaction occurred in the women who had a *Plasmodium* infection. In uninfected women, levels of Ang-1 did not alter placental barrier thickness while there is a negative association between levels of Ang-1 and placental barrier thickness in women who experienced malaria during pregnancy. A study with a larger number of samples from infected women is necessary in order to verify this observation.

Decreased levels of peripheral and placental Ang-1 have previously been associated with the occurrence of malaria in pregnancy in African women, in an area of high *P*. *falciparum* transmission and where the detrimental effects of MiP are substantial [11,38,40]. Additionally, an association between levels of Ang-1 and complement activation (responsible for vascular insufficiency in the placenta) was also observed and postulated to contribute significantly to the occurrence of low birth weight [38]. In this study, we did not observe a significant decrease in placental Ang-1 in infected women but we were able to detect an association between Ang-1 levels and placental barrier thickness in women with malaria during pregnancy, which may constitute a physical mechanism for the occurrence of vascular insufficiency. Additionally, in our study, there was no association between MiP and the occurrence of low birth weight, and so it is not possible to evaluate the real impact that this increase in thickness of the placental barrier may have. In conclusion, in this area where diagnosis and treatment of malaria are readily available and where the impact of malaria during pregnancy is mild, we were able to detect a link between decreased levels of Ang-1 and an increase in the placental barrier thickness of *Plasmodium*-infected women.

## Supporting Information

S1 Fig Effect of multiple infections on placental histopathological parameters in *P*. *vivax* infected women. The thickness of the placental barrier (**A**) and the percentage of mononuclear cells (**B**) are plotted according to the number of *P*. *vivax-*only infections detected during pregnancy. Uninfected (n = 84), one infection by *P*. *vivax* (n = 11) and more one infection by *P*. *vivax* (n = 8).
(TIF)

S2 Fig Effect of *P*. *vivax* infection status on levels of peripheral cytokines. The Ln-transformed levels of cytokines and chemokines (in pg/mL) are plotted against the *P*. *vivax* infection status during pregnancy. Uninfected (n = 12), past infection (n = 11) and current infection (n = 4).
(TIF)

S3 Fig Effect of *P*. *vivax* infection status on levels of placental cytokines. The Ln-transformed levels of cytokines and chemokines (in pg/mL) are plotted against the *P*. *vivax* infection status during pregnancy. Uninfected (n = 78), past infection (n = 15) and current infection (n = 4).
(TIF)

S1 Table Associations between placental levels of angiogenic factors and cytokines with placental histological parameters. Multivariate linear models were used to evaluate the association between angiogenic factors or cytokines with the various placental parameters of interest. A different model was run for each ln-transformed independent variable (angiogenic factors/cytokines) and the residuals tested for normality. Coefficients represent the increase or decrease in placental parameter associated with a two-fold increase of the independent variable. Boldface type represents statistical significance below the 0.05 level. All models were controlled for delivery method and gestational age at delivery.
(DOCX)

S1 Checklist STROBE checklist. (DOCX)

S1 Dataset Dataset of the study used for the analyses. (ZIP)
