# Supplementary figures and images for: Malaria in Pregnancy Interacts with and Alters the Angiogenic Profiles of the Placenta
Source: PLoS Negl Trop Dis. 2015 Jun 19;9(6):e0003824. doi: 10.1371/journal.pntd.0003824 (PMC4475015; doi:10.1371/journal.pntd.0003824)

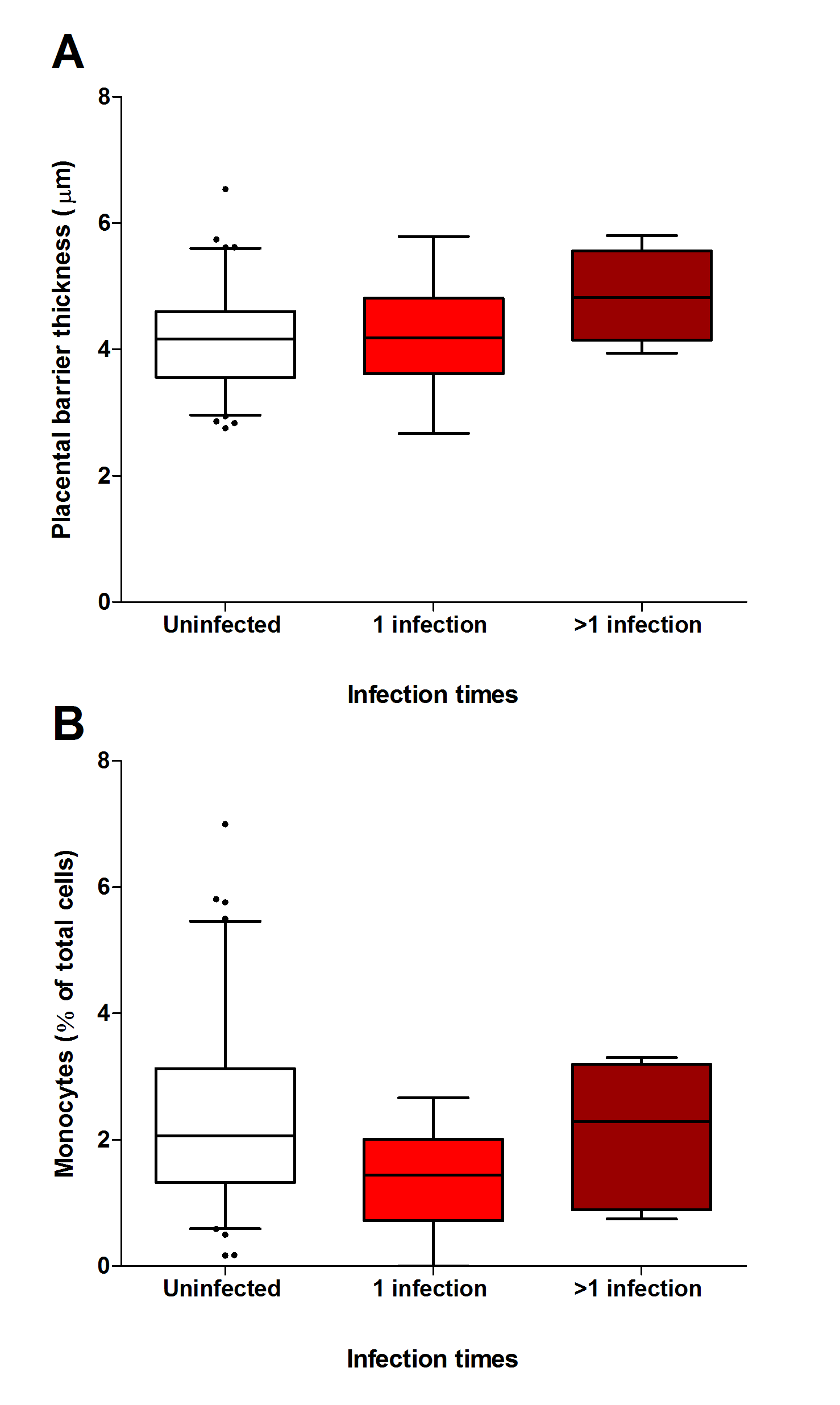

Supplement: S1 Fig — The thickness of the placental barrier (A) and the percentage of mononuclear cells (B) are plotted according to the number of P. vivax-only infections detected during pregnancy. Uninfected (n = 84), one infection by P. vivax (n = 11) and more one infection by P. vivax (n = 8). (TIF) [file pntd.0003824.s001.tif]

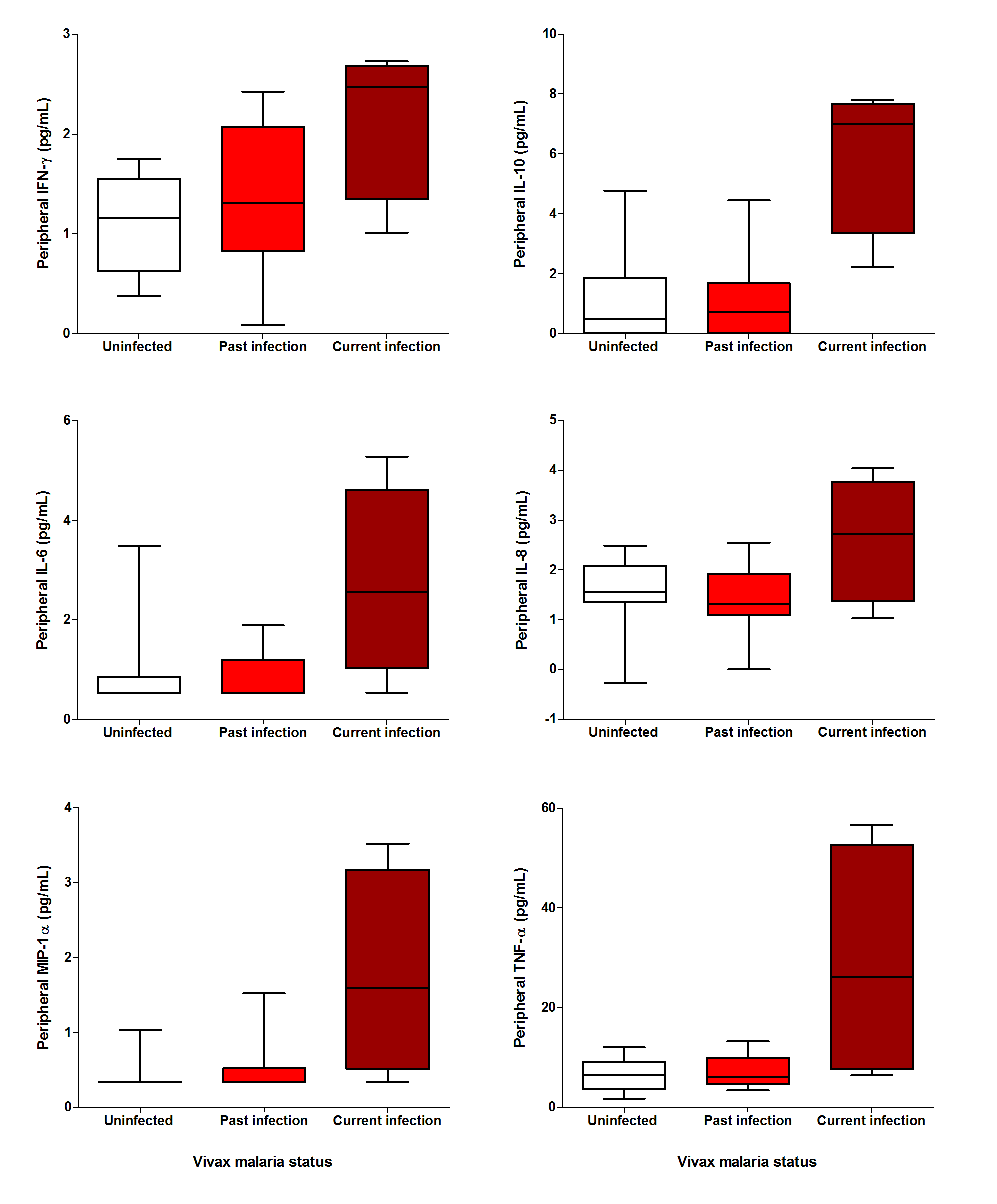

Supplement: S2 Fig — The Ln-transformed levels of cytokines and chemokines (in pg/mL) are plotted against the P. vivax infection status during pregnancy. Uninfected (n = 12), past infection (n = 11) and current infection (n = 4). (TIF) [file pntd.0003824.s002.tif]

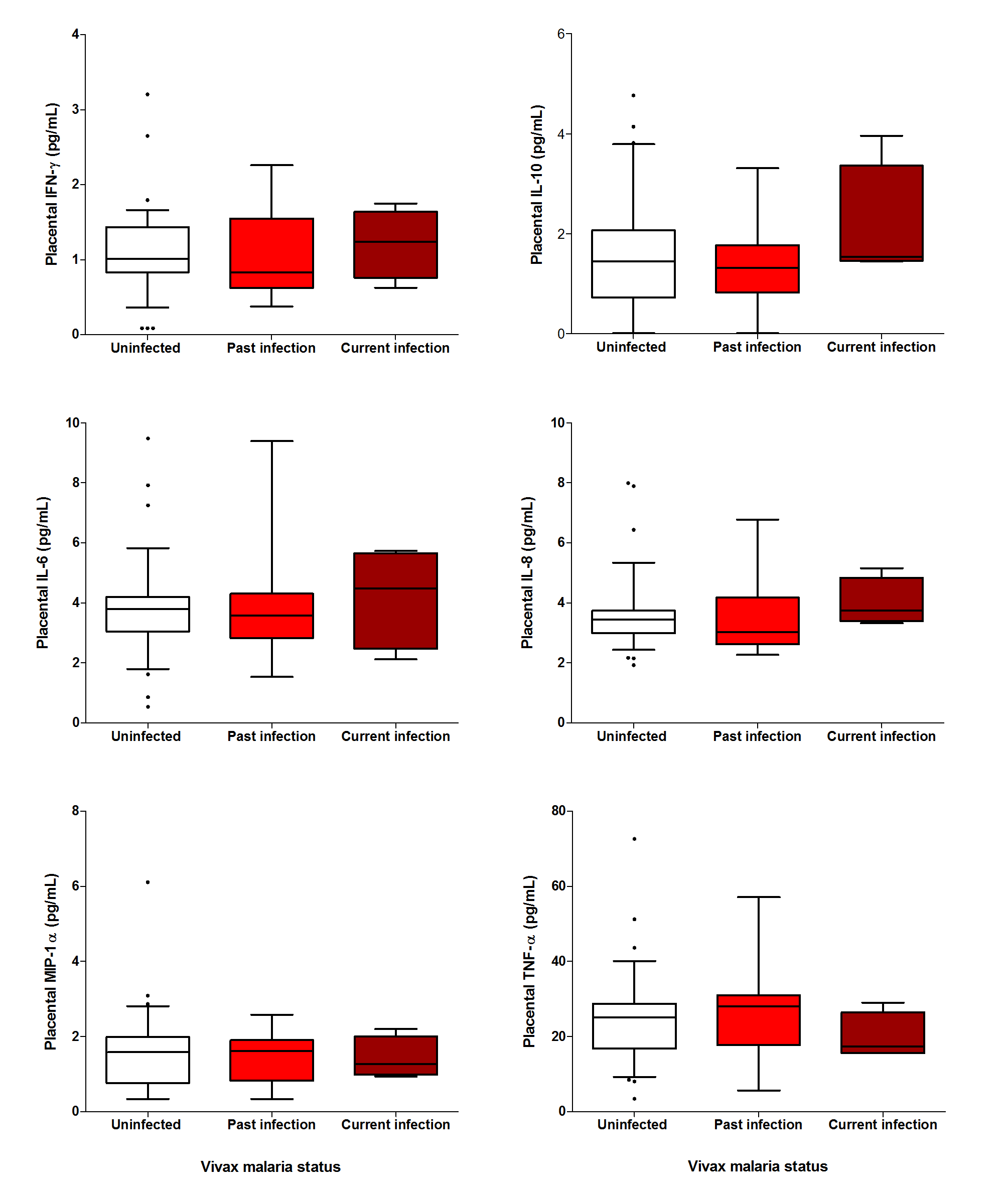

Supplement: S3 Fig — The Ln-transformed levels of cytokines and chemokines (in pg/mL) are plotted against the P. vivax infection status during pregnancy. Uninfected (n = 78), past infection (n = 15) and current infection (n = 4). (TIF) [file pntd.0003824.s003.tif]
