# Supplementary material for: Malaria in Pregnancy Interacts with and Alters the Angiogenic Profiles of the Placenta
Source: PLoS Negl Trop Dis. 2015 Jun 19;9(6):e0003824. doi: 10.1371/journal.pntd.0003824 (PMC4475015; doi:10.1371/journal.pntd.0003824)
Supplement: S1 Table — Multivariate linear models were used to evaluate the association between angiogenic factors or cytokines with the various placental parameters of interest. A different model was run for each ln-transformed independent variable (angiogenic factors/cytokines) and the residuals tested for normality. Coefficients represent the increase or decrease in placental parameter associated with a two-fold increase of the independent variable. Boldface type represents statistical significance below the 0.05 level. All models were controlled for delivery method and gestational age at delivery. (DOCX) [file pntd.0003824.s004.docx]

S1 Table. Associations between placental levels of angiogenic factors and cytokines with placental histological parameters.

|  | **Placental weight (g)** | **Syncytial knots (%)** | **Barrier thickness (μm)** | **Mononuclear cells (%)** |
| --- | --- | --- | --- | --- |
| **Angiogenic factors** |  |  |  |  |
| **Angiopoietin-1** | -36.34 [-69.76, -2.91] | 0.24 [-1.63, 2.11] | -0.30 [-0.60, -0.01] | -0.25 [-0.80, 0.29] |
| **Angiopoietin-2** | -10.35 [-34.14, 13.44] | -0.09 [-1.40, 1.23] | 0.30 [0.10, 0.51] | -0.21 [-0.60, 0.18] |
| **Tie-2** | 10.32 [-57.29, 77.93] | -1.09 [-4.82, 2.63] | -0.02 [-0.62, 0.59] | 0.31 [-0.79, 1.42] |
| **Ratio Tie-2/Angiopoietin-1** | 32.22 [1.56, 62.88] | -0.42 [-2.13, 1.29] | 0.26 [-0.02, 0.53] | 028 [-0.22, 0.79] |
| **Cytokines** |  |  |  |  |
| **IFN-γ** | -3.82 [-35.10, 27.47] | 0.56 [-1.16, 2.27] | 0.16 [-0.11, 0.44] | -0.17 [-0.67, 0.33] |
| **IL-10** | -9.87 [-26.44, 6.71] | -0.68 [-1.59, 0.23] | 0.04 [-0.10, 0.19] | 0.17 [-0.10, 0.44] |
| **IL-6** | -2.10 [-14.77, 10.57] | -0.45 [-1.14, 024] | 0.12 [0.01, 0.23] | 0.02 [-0.18, 0.22] |
| **IL-8** | -13.45 [-30.84, 3.93] | 0.05 [-0.91, 1.02] | 0.16 [0.01, 0.31] | 0.03 [-0.26, 0.31] |
| **MIP1-α** | 1.87 [-17.97, 21.72] | -0.46 [-1.55, 0.63] | -0.01 [-0.18, 0.17] | 0.18 [-0.13, 0.50] |
| **TNF-α** | 0.89 [-0.78, 2.56] | -0.00 [-0.09, 0.09] | -0.00 [-0.02, 0.01] | 0.01 [-0.02, 0.03] |

Multivariate linear models were used to evaluate the association between angiogenic factors or cytokines with the various placental parameters of interest. A different model was rum for each ln-transformed independent variable (angiogenic factors/cytokines) and the residuals tested for normality. Coefficients represent the increase or decrease in placental parameter associated with a two-fold increase of the independent variable. Boldface type represents statistical significance below the 0.05 level. All models were controlled for delivery method and gestational age at delivery.
